# Prognosis in Congenital Heart Disease

**Published:** 1908-11-21

**Authors:** 


### PROGNOSIS IN CONGENITAL HEART DISEASE.

Scientifically speaking, there are scores of different forms of congenital heart disease. Clinically speaking, on the other hand, there are only three—namely, patent septum ventriculorum, pulmonary stenosis, and patent ductus arteriosus. The first of these may occur either by itself or in association with either of the other two.

It is always very hard to estimate the prognosis in a young case of congenital heart disease. In some patients it is absolutely bad; in others only fair; whilst in a few it is so good that the patient may live an ordinary life without suffering any inconvenience at all. It is to emphasise the latter point that we quote the following case of patent septum ventriculorum in a woman of fifty-five.

She had been married and had borne three children, all of whom she had suckled. Her pregnancies had been uneventful. Indeed, after the illnesses of childhood were past the patient had not been ill at all until she was fifty-five. There was a very loud bruit, typical of patent septum ventriculorum, systolic in time, superficial, best heard at the inner end of the third left intercostal space, and radiating thence widely over the front and back of the chest. She finally consulted a medical man for gastric symptoms, and the cardiac lesion was discovered unexpectedly in the course of the routine examination.

It will be supposed that there can have been only a minute hole in the interventricular septum in this case, causing a great noise but little real disturbance of the circulation. This is very probable; but the degree of the lesion, and hence the prognosis, could scarcely have been determined from the physical signs alone. The fact of survival is the chief evidence that the functional disturbance was slight; the point is that survival is possible, notwithstanding the presence of well-marked physical signs of congenital heart disease.

